# Supplementary figures and images for: Uncovering the Genetic Structure of the Sekler Population in Transylvania Through Genome-Wide Autosomal Data
Source: Genes (Basel). 2025 Dec 29;17(1):30. doi: 10.3390/genes17010030 (PMC12840628; doi:10.3390/genes17010030)

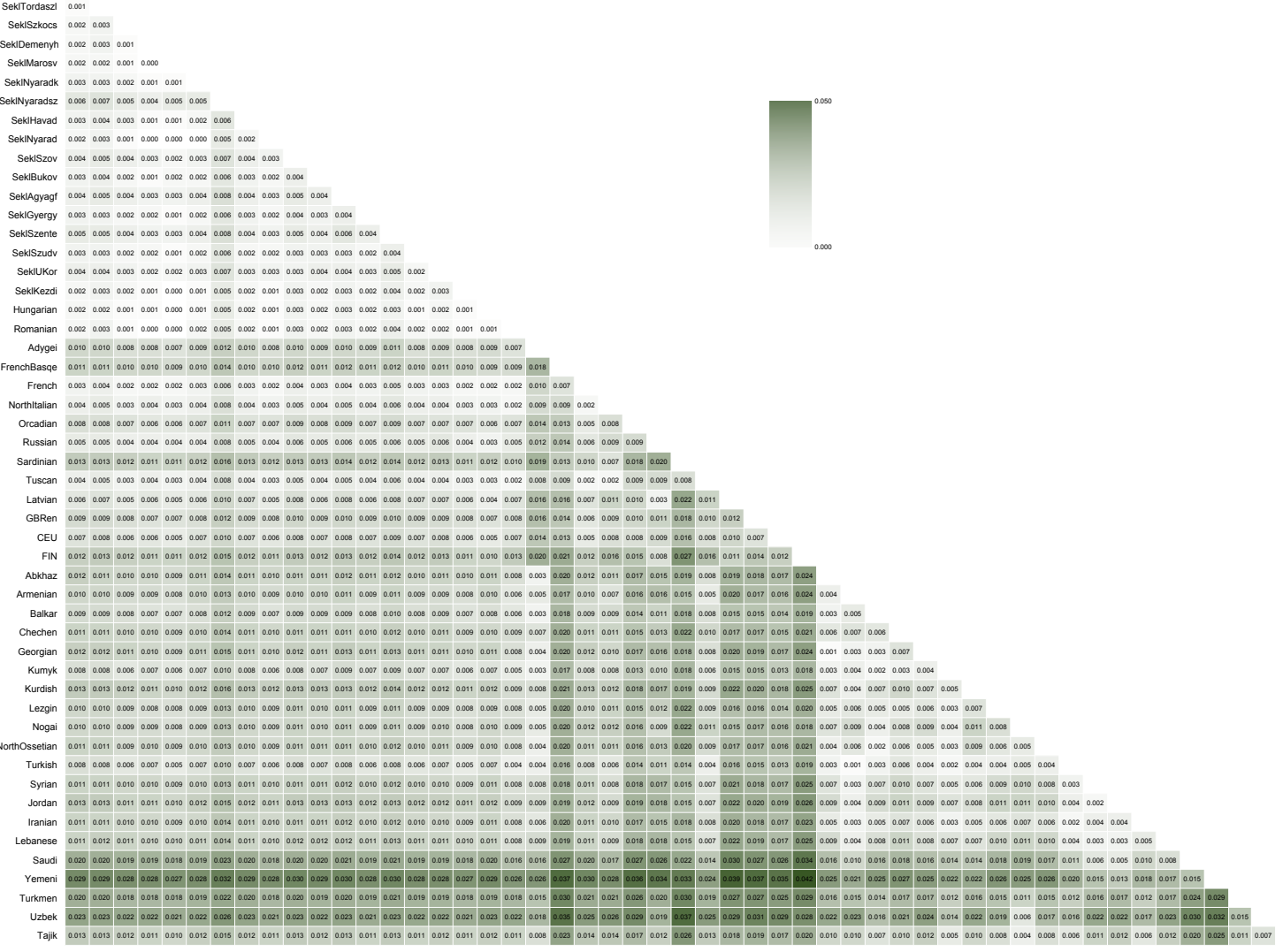

Supplement: Supplementary file 1 [file genes-17-00030-s001.zip › Figure_S5.pdf]

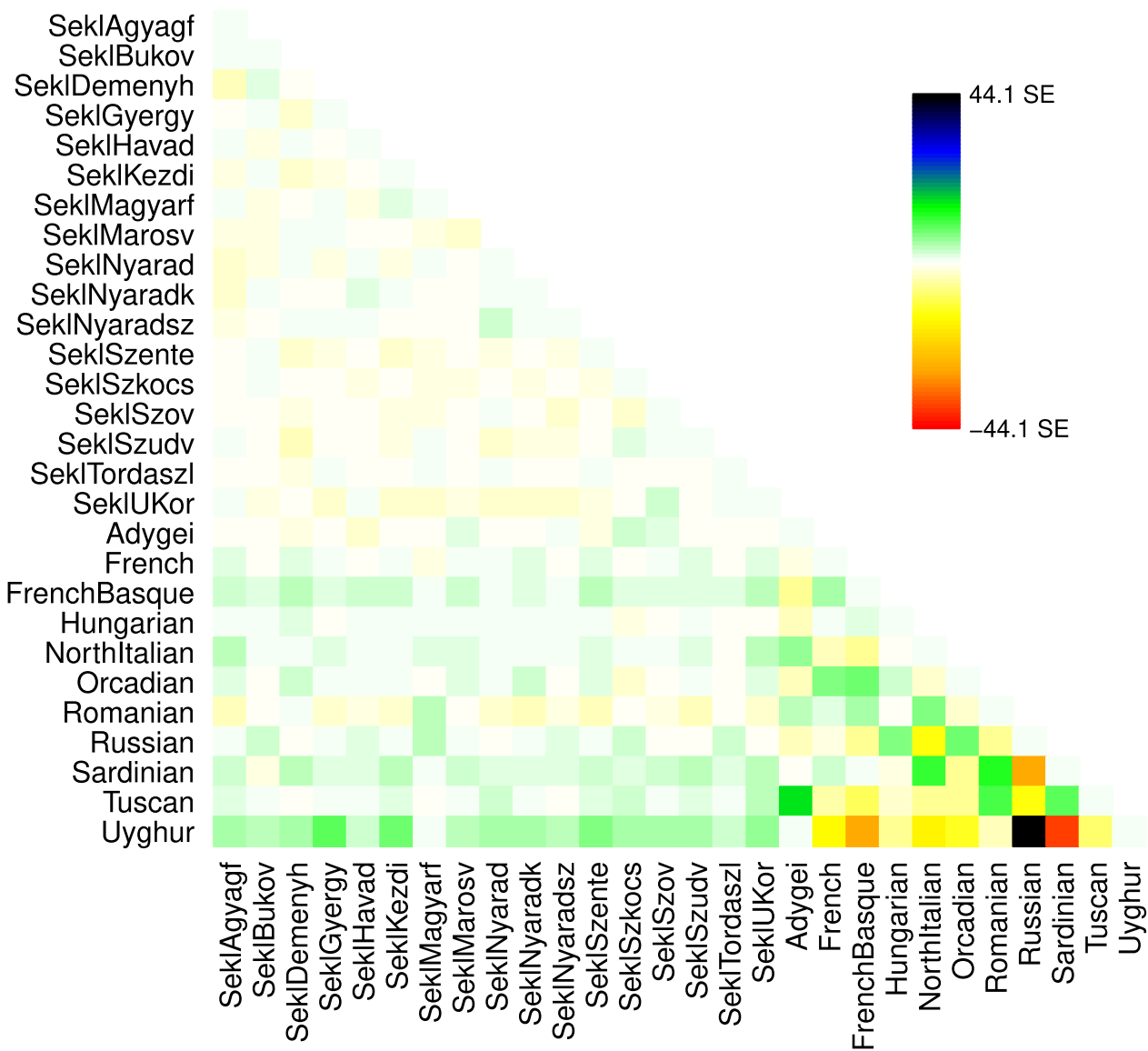

Supplement: Supplementary file 1 [file genes-17-00030-s001.zip › Figure_S4.pdf]

K=10

K=9

K=8

K=7

K=6

K=5

K=4

K=3

K=2

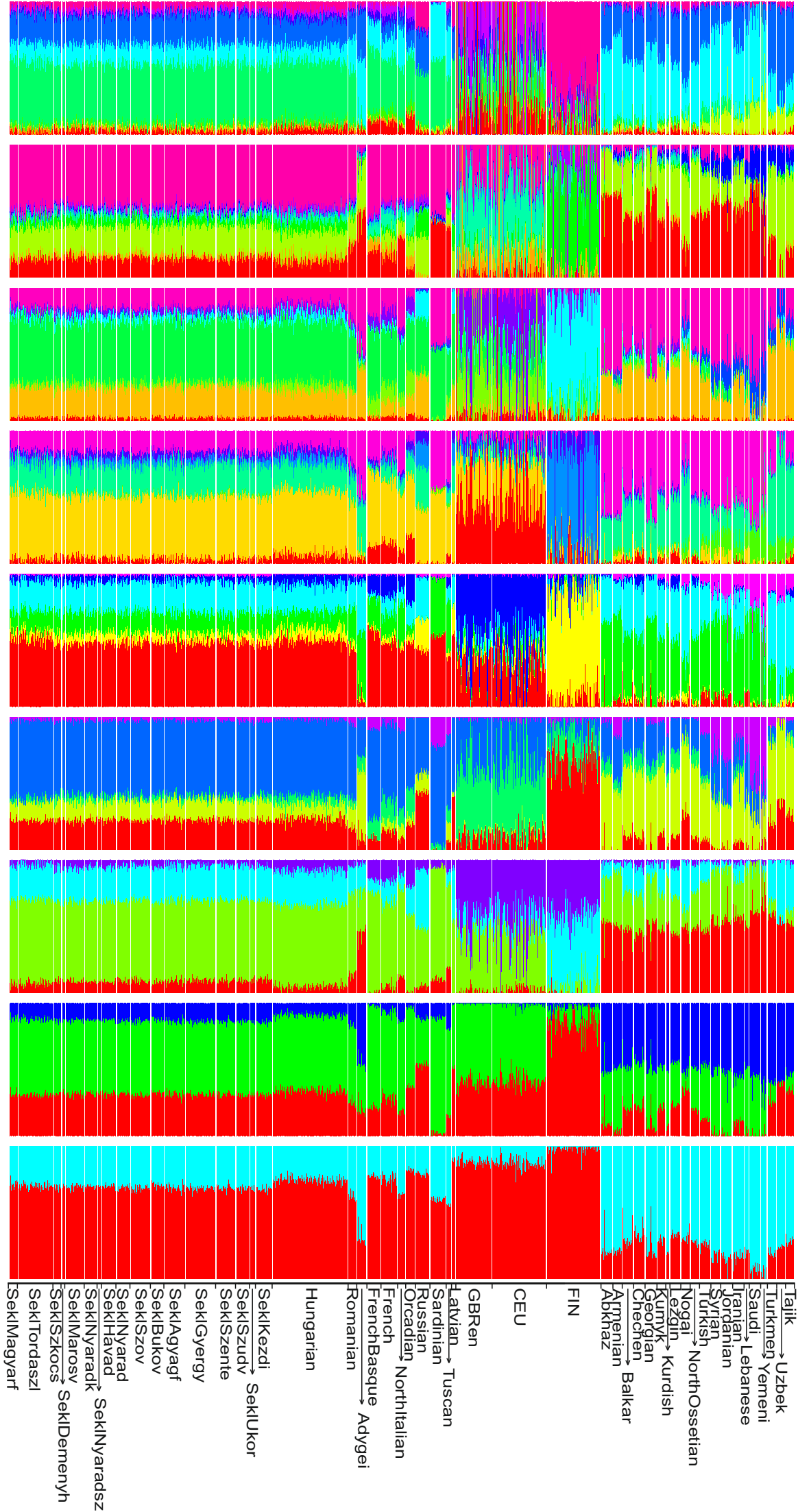

Supplement: Supplementary file 1 [file genes-17-00030-s001.zip › Figure_S3.pdf]

## Scree plot of Eigenvalues calculated by SMARTPCA

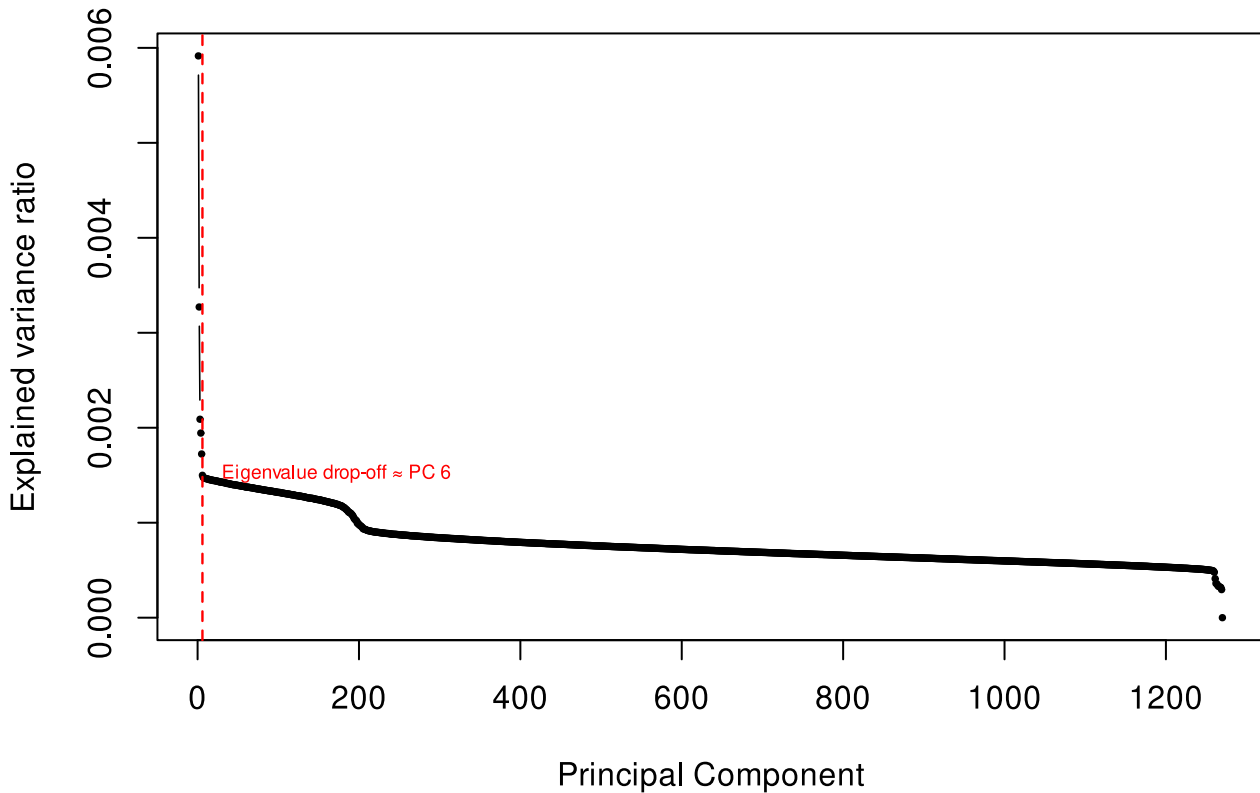

Supplement: Supplementary file 1 [file genes-17-00030-s001.zip › Figure_S2.pdf]

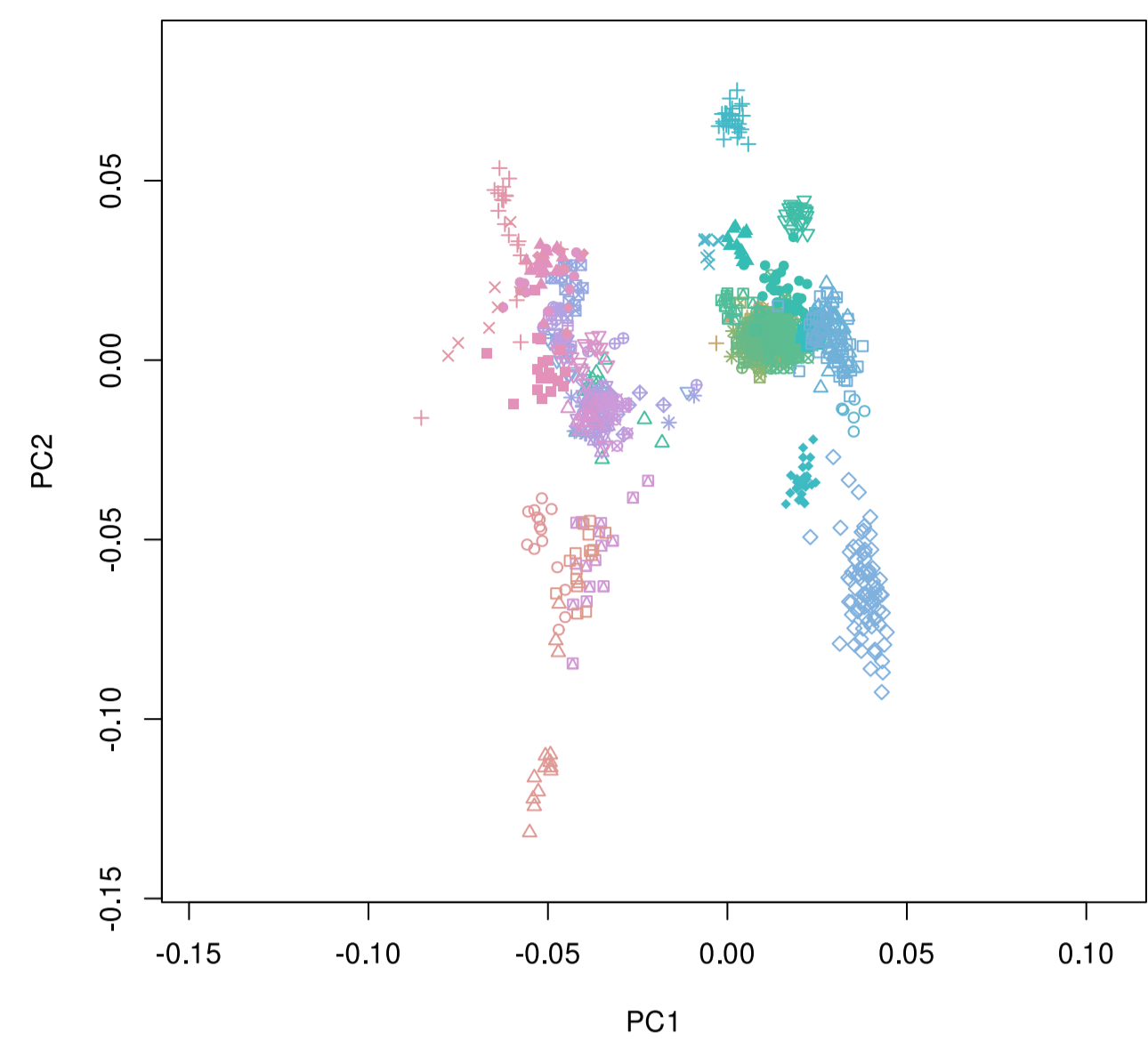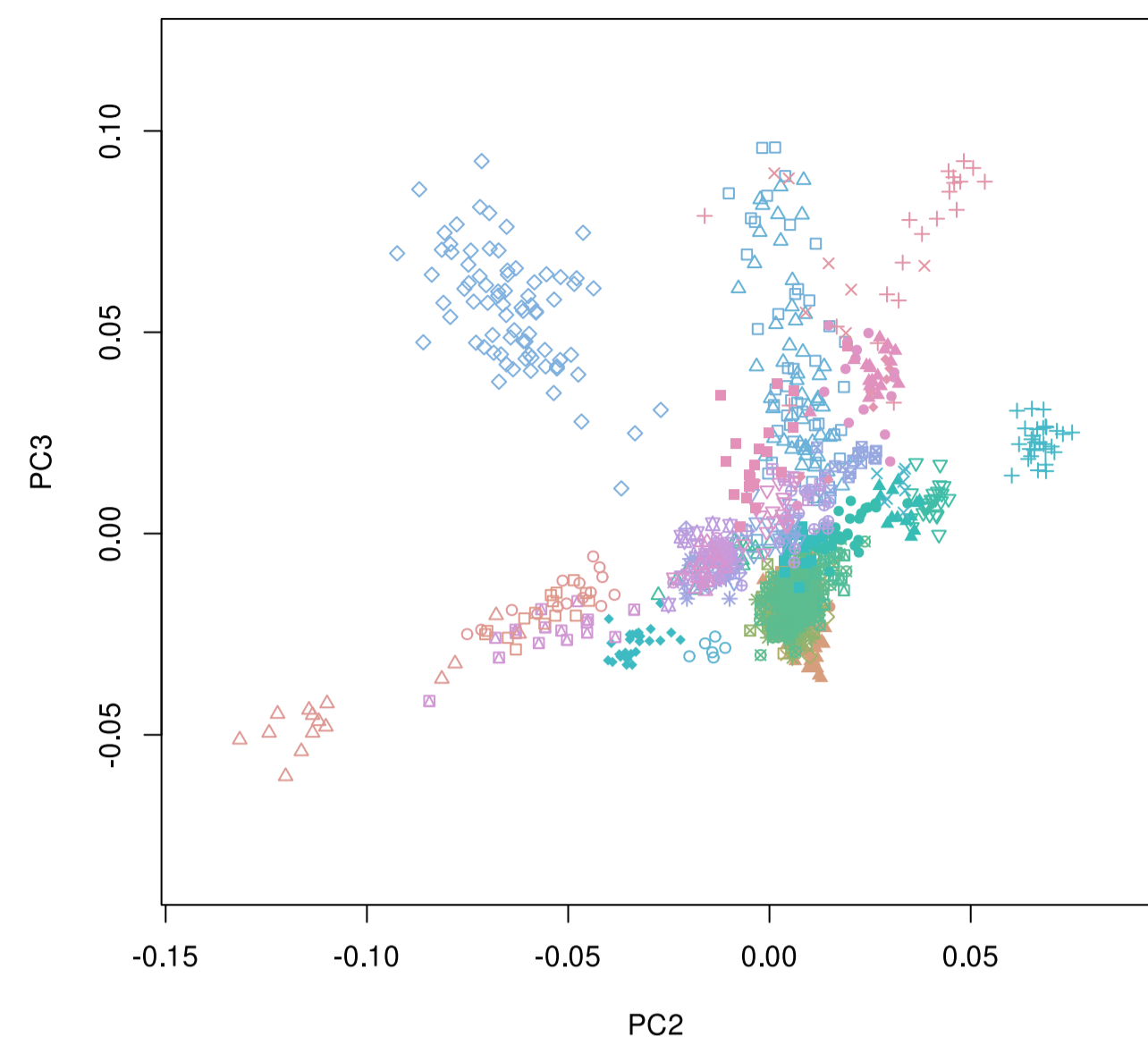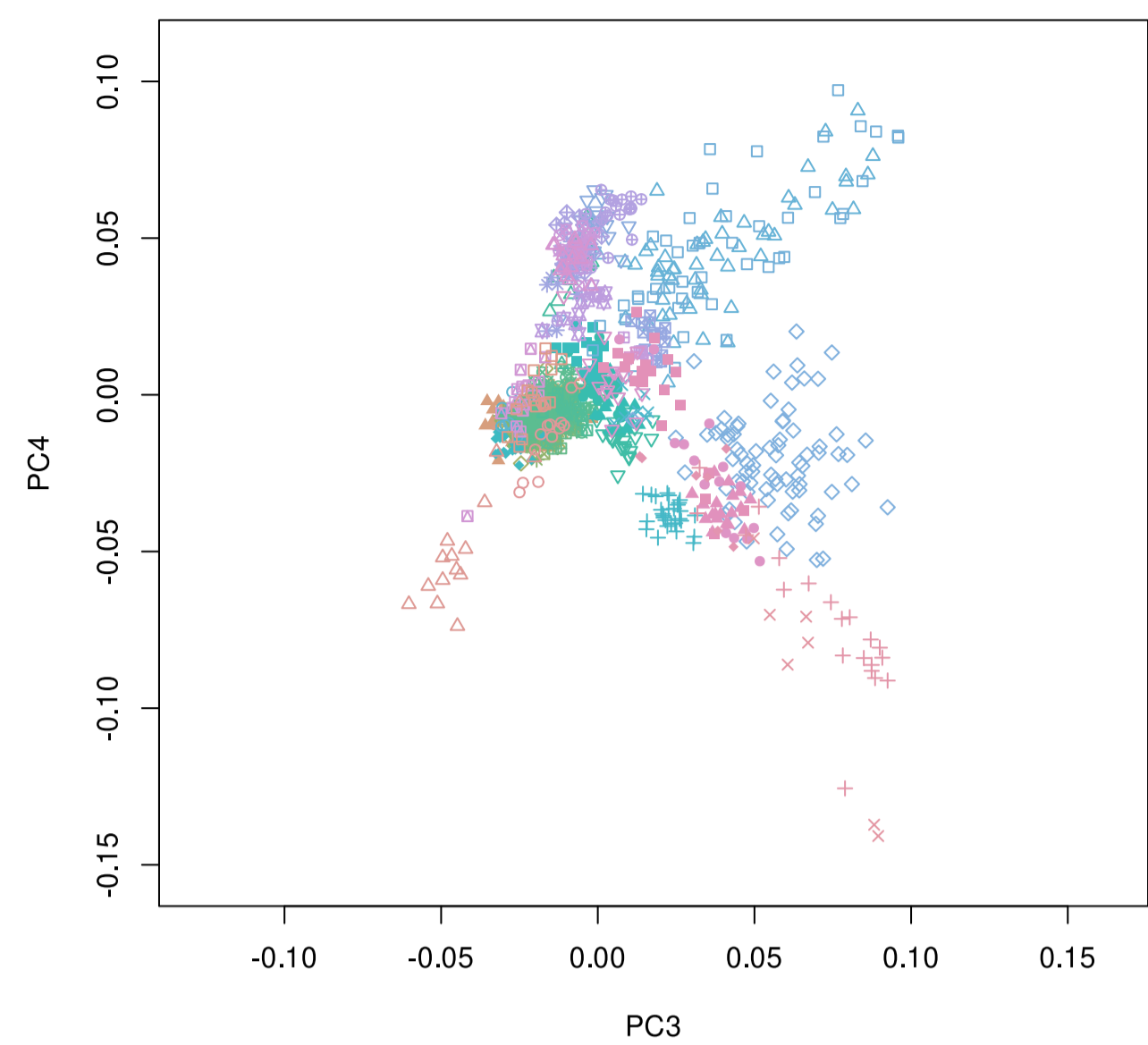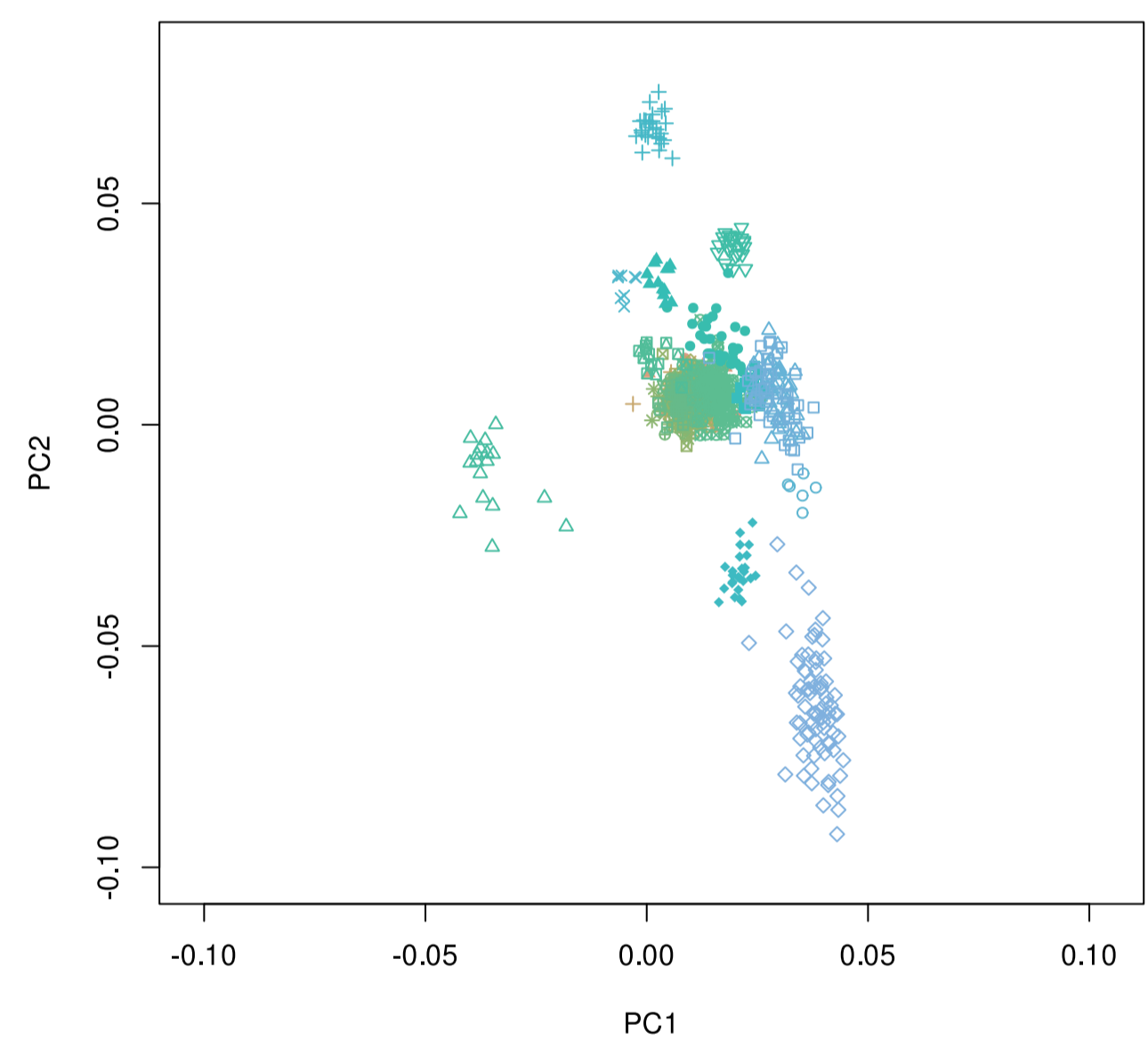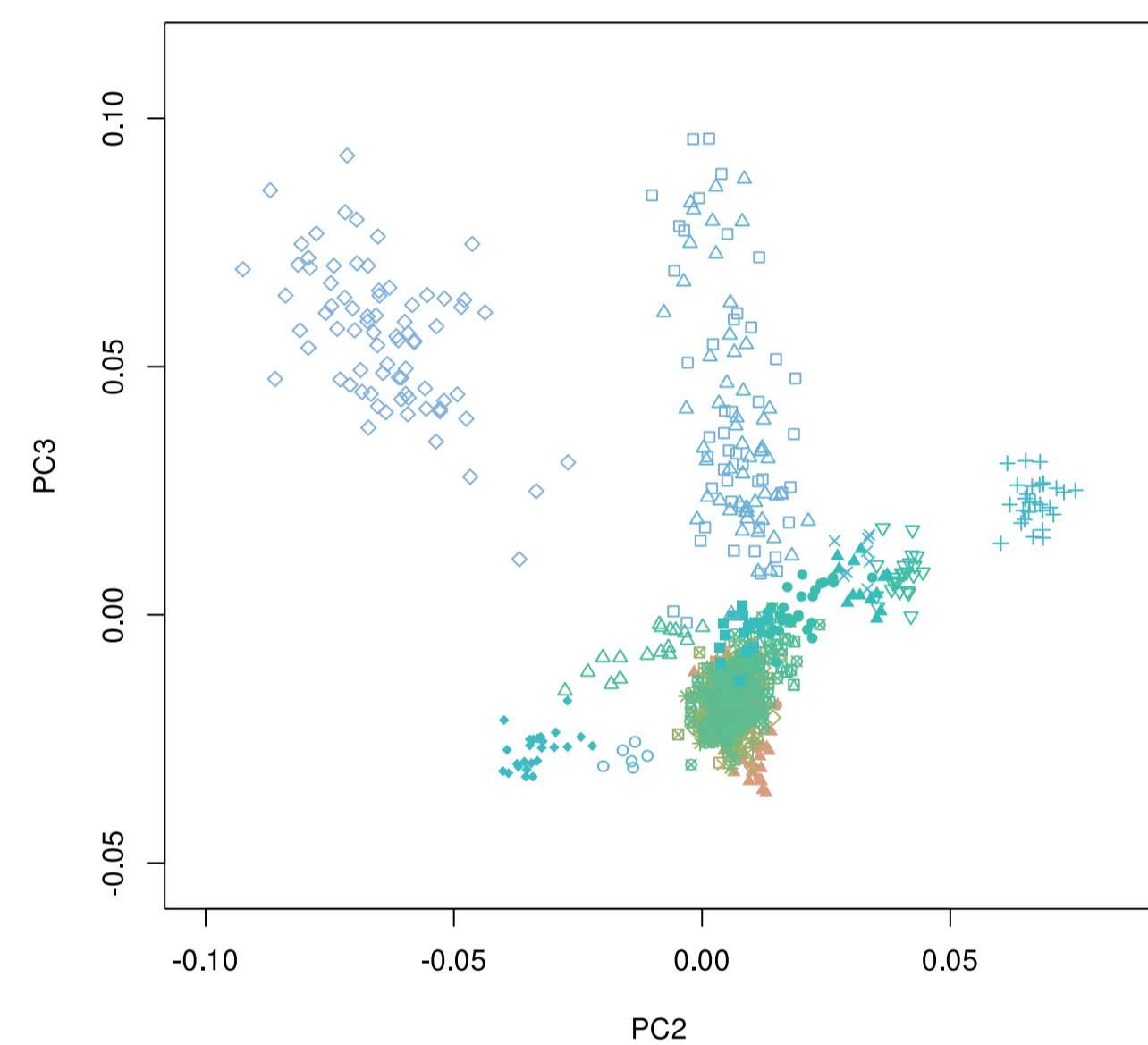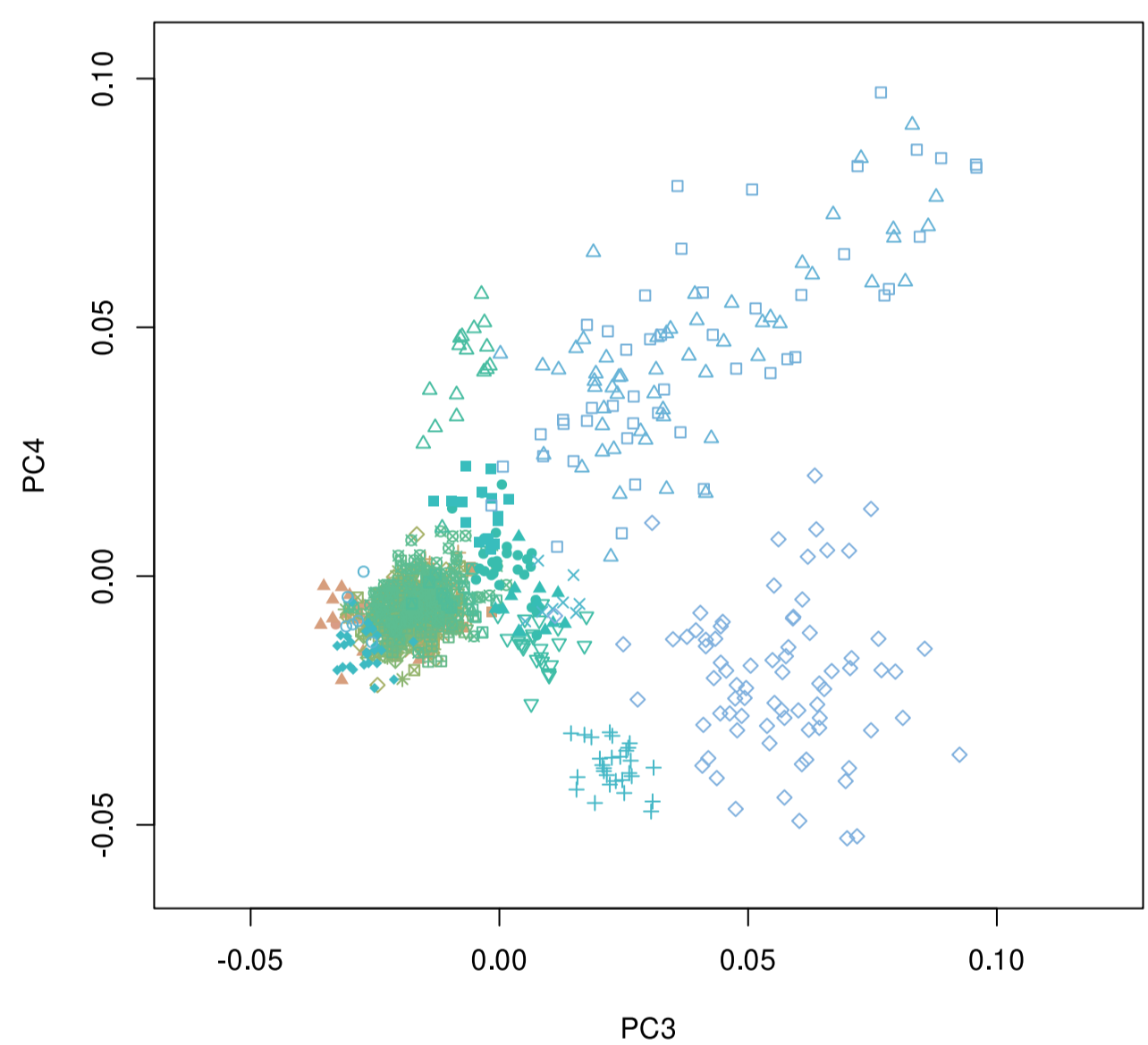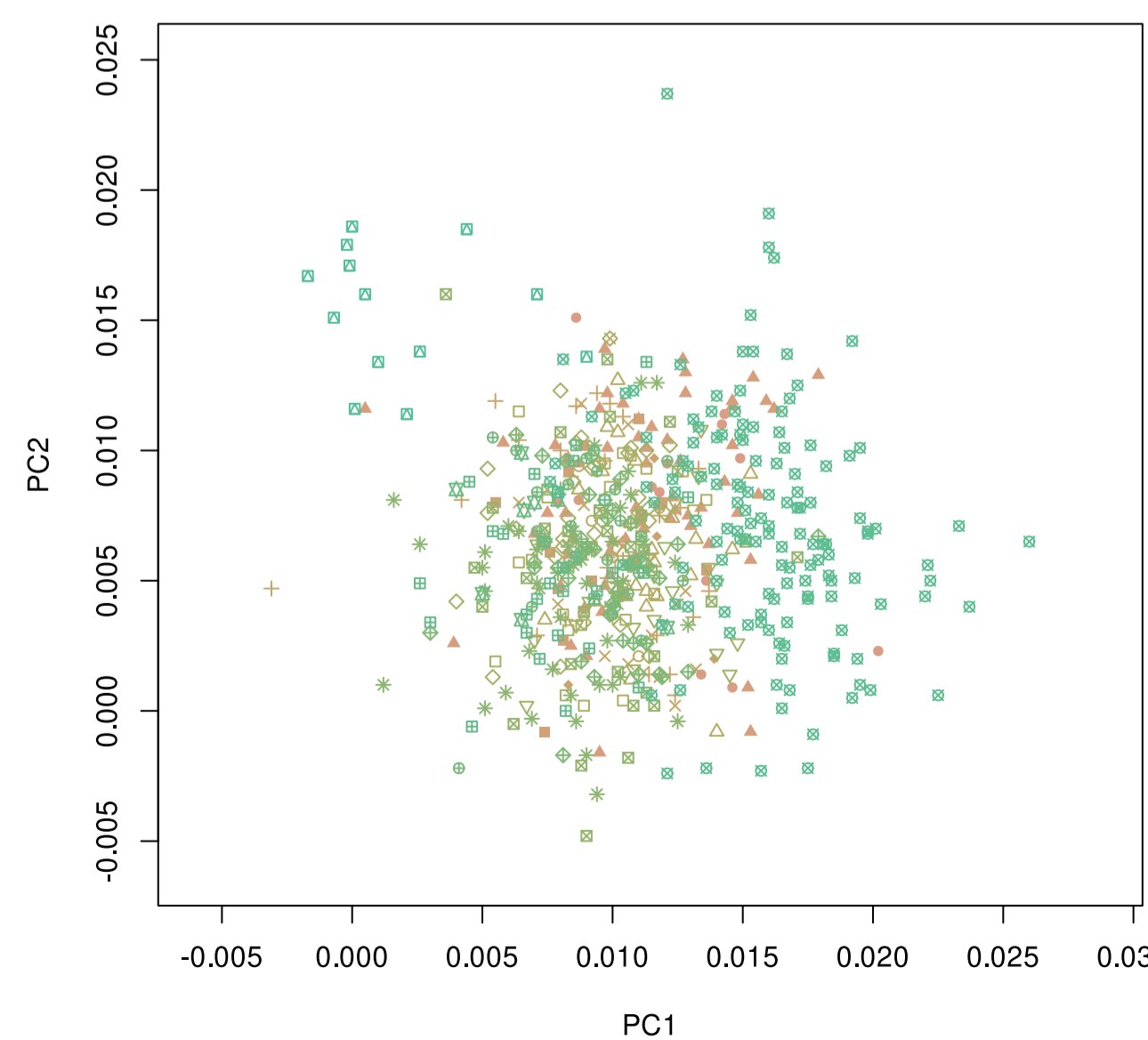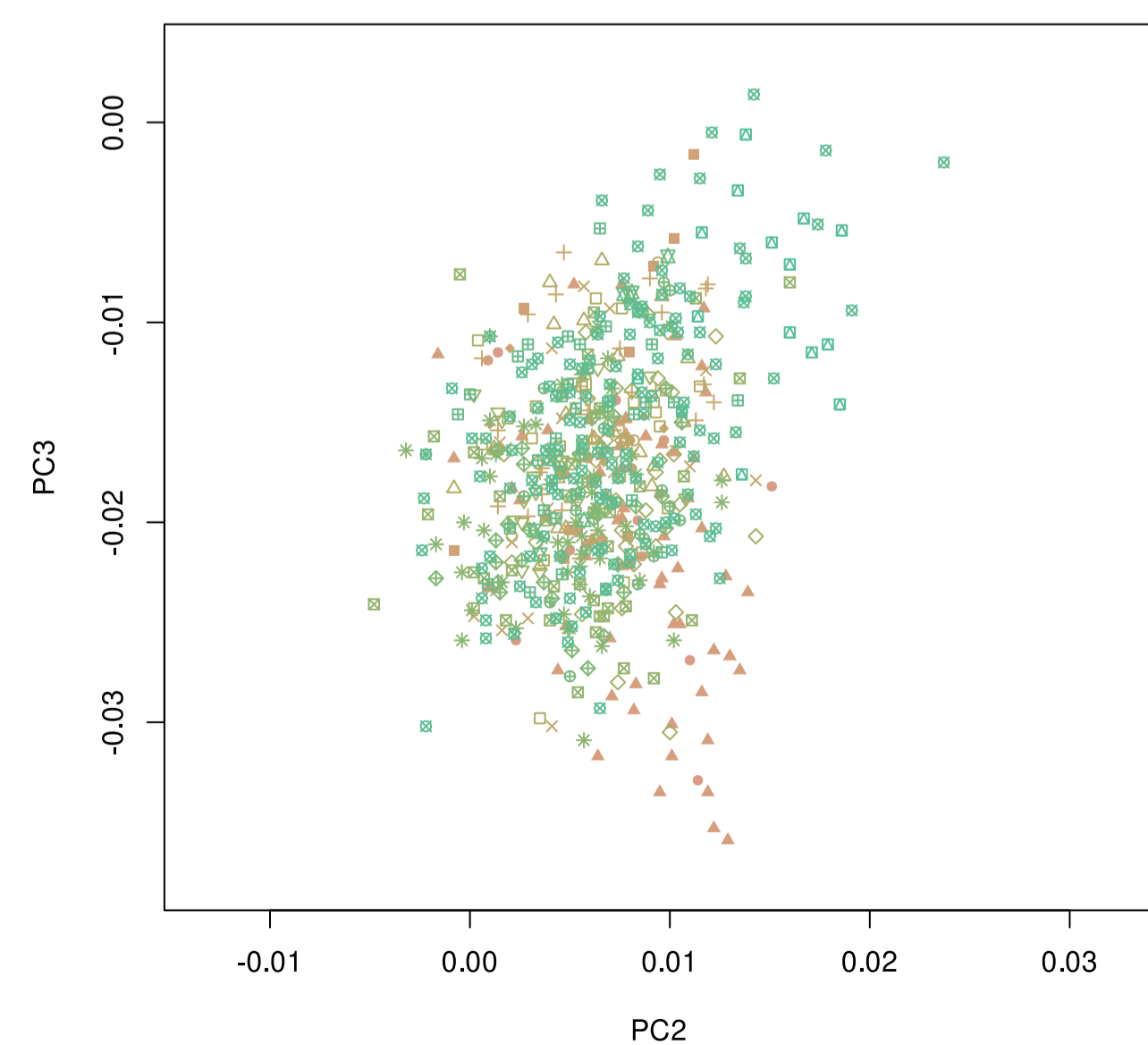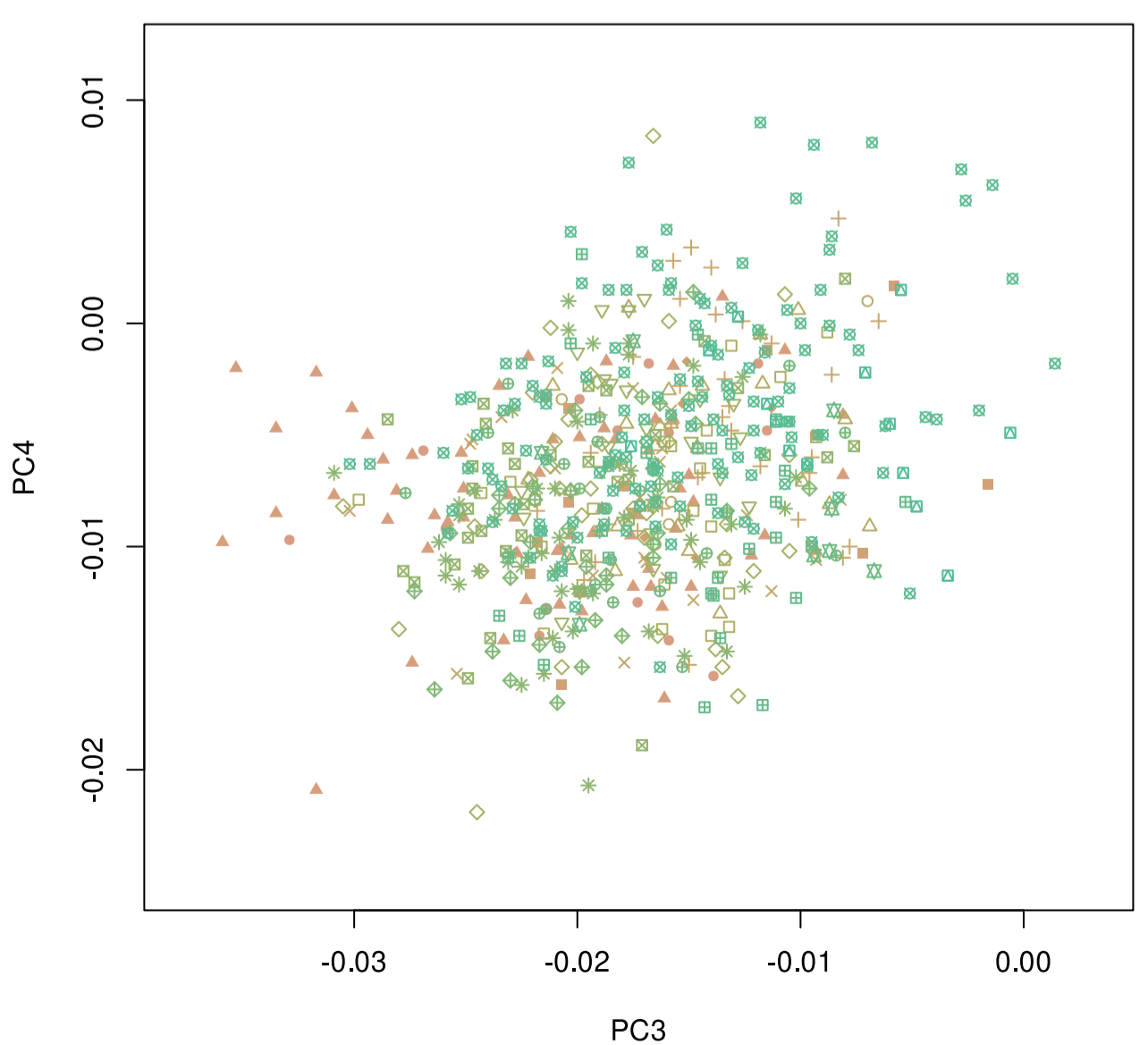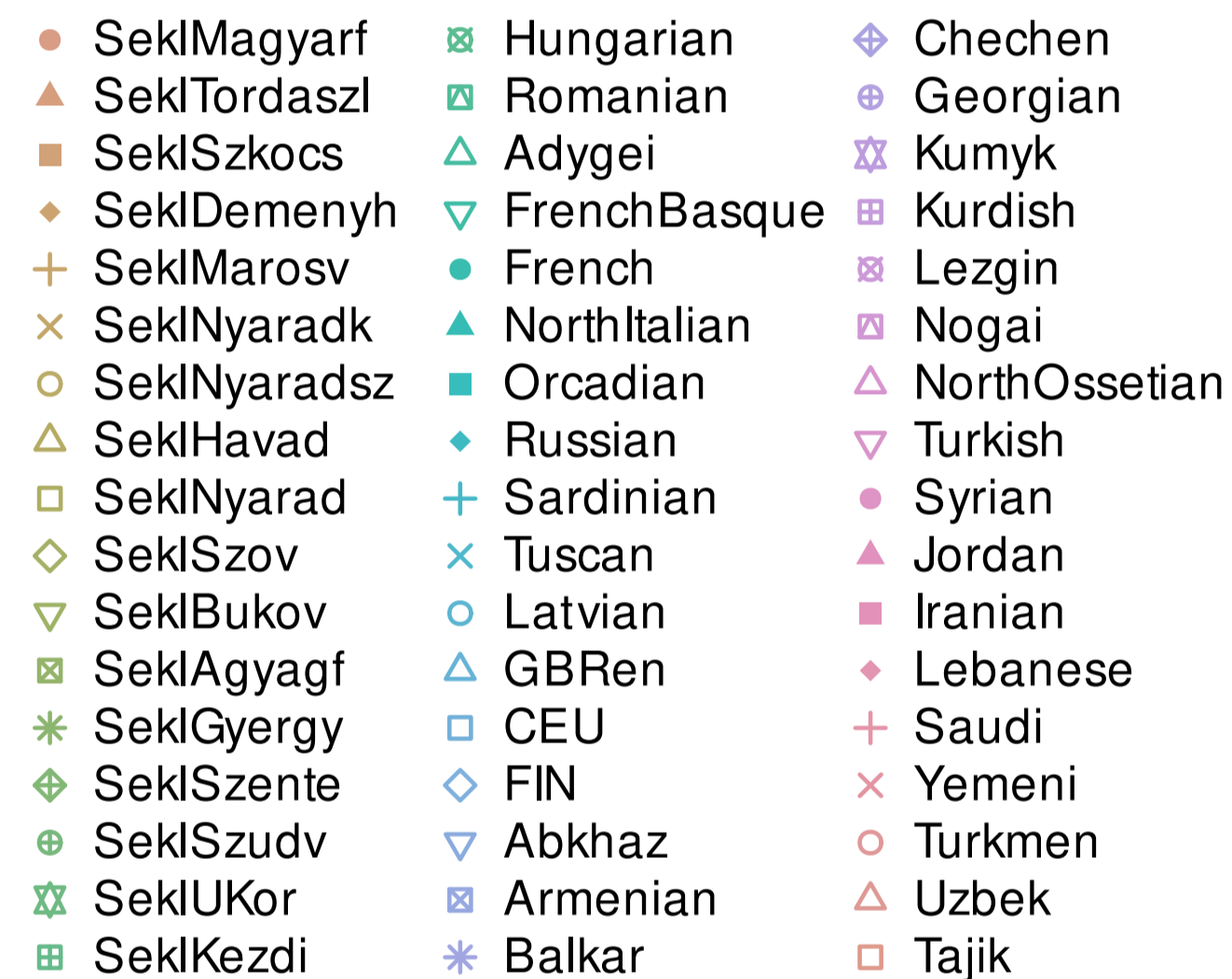

Supplement: Supplementary file 1 [file genes-17-00030-s001.zip › Figure_S1.pdf]
